# Formal Analysis of Trust and Reputation for Service Composition in IoT

**DOI:** 10.3390/s23063192

**Published:** 2023-03-16

**Authors:** Abdelmuttlib Ibrahim Abdalla Ahmed, Siti Hafizah Ab Hamid, Abdullah Gani, Ahmed Abdelaziz, Mohammed Abaker

**Affiliations:** 1 Department of Computer System and Technology, Faculty of Computer Science and Information Technology, University of Malaya, Kuala Lumpur 50603, Malaysia; abdelmuttlib@siswa.um.edu.my; 2 Department of Software Engineering, Faculty of Computer Science and Information Technology, University of Malaya, Kuala Lumpur 50603, Malaysia; 3 Faculty of Computing and Informatics, Universiti Malaysia Sabah (UMS), Kota Kinabalu 88400, Malaysia; 4 Khawarizmi International College, Abu Dhabi P.O. Box 25669, United Arab Emirates; 5 Department of Computer Science, Applied College, King Khalid University, Muhayil 61913, Saudi Arabia

**Keywords:** service, service composition, IoT

## Abstract

The exponential growth in the number of smart devices connected to the Internet of Things (IoT) that are associated with various IoT-based smart applications and services, raises interoperability challenges. Service-oriented architecture for IoT (SOA-IoT) solutions has been introduced to deal with these interoperability challenges by integrating web services into sensor networks via IoT-optimized gateways to fill the gap between devices, networks, and access terminals. The main aim of service composition is to transform user requirements into a composite service execution. Different methods have been used to perform service composition, which has been classified as trust-based and non-trust-based. The existing studies in this field have reported that trust-based approaches outperform non-trust-based ones. Trust-based service composition approaches use the trust and reputation system as a brain to select appropriate service providers (SPs) for the service composition plan. The trust and reputation system computes each candidate SP’s trust value and selects the SP with the highest trust value for the service composition plan. The trust system computes the trust value from the self-observation of the service requestor (SR) and other service consumers’ (SCs) recommendations. Several experimental solutions have been proposed to deal with trust-based service composition in the IoT; however, a formal method for trust-based service composition in the IoT is lacking. In this study, we used the formal method for representing the components of trust-based service management in the IoT, by using higher-order logic (HOL) and verifying the different behaviors in the trust system and the trust value computation processes. Our findings showed that the presence of malicious nodes performing trust attacks leads to biased trust value computation, which results in inappropriate SP selection during the service composition. The formal analysis has given us a clear insight and complete understanding, which will assist in the development of a robust trust system.

## 1. Introduction

In SOA-IoT, the system decomposes the request and finds suitable services and service providers (SPs) to compose a holistic service, which fits the business process’s specified requirements. Service and SP selection is a critical task during the service composition process. Different approaches have been followed in performing this task: trust-based and non-trust-based [1,2]. The existing studies in this field have shown that trust-based methods outperform non-trust-based ones [2]. Trust-based service composition applications use a trust and reputation system as a brain to compute the trust values of each candidate SP. The system selects the candidate with the highest trust value as the SP for each step of the service composition plan. The trust system calculates the trust values based on self-observation (direct trust) and user feedback (recommendation). However, trust systems in IoT trust-based service composition may not always estimate the actual or accurate trust value. As a result, the system may select inappropriate SPs, which leads to a low-quality service composition for IoT users.

A good trust system can boost the reliability of the interactions and the cooperation among the entities of a collaborative IoT system, particularly in exchanging and aggregating self-observations, computing the trust values of service providers, and sharing these values [3,4,5,6,7]. For example, a trust-based system is based on the service’s ability to cooperatively process the sensed data on distributed, centralized, or cloud-based reputation systems [3,4,8,9,10,11]. The robustness of the trust system represents the ability of the system to sustain the accuracy of the computed trust value under heterogeneity and the presence of malicious entities [12]. These malicious entities perform different types of trust attacks, and these attacks involve several misleading behaviors, which result in the manipulation of trust values [13,14,15,16,17,18,19,20]. Several experimental solutions have been proposed to deal with these service composition issues in the IoT. However, a formal method for trust-based service composition in the IoT is still lacking. The existing studies that have focused on formal methods for service composition are summarized in Table 1.

The remaining sections in this article are organized as follows: Section 2 explains the formal method and the semantics of HOL. Section 3 focuses on the formal representation of trust-based SOA-IoT. Section 4 provides a formal representation of the trust system. Section 5 presents the execution semantics of trust-based service composition. Section 6 highlights the performance metrics of the trust system. Section 7 presents a case study of trust-based service composition in the IoT. Finally, Section 8 concludes the article.

## 2. Formal Methods

In this section, we briefly explain the formal methods that we used during our analysis of the trust-based service composition problems in IoT systems.

### 2.1. Formal Definitions

Here, we briefly discuss higher logic (HOL) [25]. Next, we provide an argument in our discussion, which includes the details of how HOL can describe the trust system of a trust-based SOA-IoT. HOL is a set of formal information representations for a particular application area. HOL has the best expressive power, compared to first- and second-order predicate logic. The brain behind HOL is typically definable and uses proficient methods.

HOL includes atomic formulas, which are produced from a set (L) of non-logical constants, which can help in distinguishing individual constants, relation signs, and function symbols. The HOL is obtained by turning atomic formulas into an inductive definition, allowing for the formation of more complicated types, and considering quantifiers for all such types.

### 2.2. HOL Syntax and Semantics

Higher logic has several options for representing and analyzing different types of studies. We used higher logic methods that extend the HOL programming approach. This approach describes an analog of the Horn clause with a rich HOL, which was introduced by Church in simple type theory [26]. HOL has two types of symbols: logical symbols and non-logical symbols. The logical symbols used in this article and their meaning are presented in Table 2.

Table 3 defines a formal representation of trust-based SOA-IoT by using HOL symbols and interaction ways between IoT entities.

## 3. Formal Representation of Trust-Based IoT System

This section provides a description and a formal representation of a trust-based IoT system, in terms of the transaction component trust model and the entity behaviors.

### 3.1. Transactions in SOA-IoT System

**Definition 1.**  *The nodes in an SOA-IoT system are three-tuple*, 𝕋= (Γ, Ψ, S[i]), *where* Γ *denotes a non-empty set of SRs*; Ψ *denotes a non-empty set of SPs; and* q ∈ *Q denotes the quality of service that is provided by a SP*.

(1) ∀ Π≝∃Γ∧∃Ψ∧∃ S ∃q

**Theorem 1.**  *By assuming the definition* (Π) *in Equation (1)* *was not biased for all transactions, we concluded that the statements in Equation (2) were correct for all transactions in the SOA-IoT system*.

(2) ∀ Π→∃Γ ∃Ψ∧∃ S∧∃q

**Proof of Theorem 1.**  The statements in Theorem 1 for the SOA-IoT transactions are non-trivial, as they always evaluate to be a universal truth for all occurrences of SOA-IoT transactions when

$$\left(\infty <\Gamma >0\right)\;\left(\infty <\Psi >0\right)\;\left(\infty <S[i]>0\right)\;\left(\infty <q>0\right)$$

Because we defined the SOA-IoT transaction and demonstrated that it was a non-empty set of four-tuples (Γ, Ψ, S, q), we could go further in defining the trust model. This is used as an integral part of service composition and SP selection during SOA-IoT transactions. □

### 3.2. Formal Representation Trust-Based Service Composition

We modeled an atomic web service as a unit of trust-based service composition in the IoT, following the workflow paradigm. We emphasized trust-based SP selection as it is the main task in each phase of the service composition (workflow). We formally defined IoT services as follows:

**Definition 2.**  *IoT services* (Ζ) *are identified and performed as* Ζ(ℕ,τ), *whereas* (ℕ) *represents service details, and* (τ) *denotes the trust value of the SP, as follows*:

(3) Ζ≝∃ℕ∧∃τ

The service information (ℕ) was elaborated with some information, such as the service ID, the SP ID, and the QoS. The trust value is detailed in Definition 3.

**Definition 3.**  *The trust system calculates the trust value*  (τ) *of every SR for the candidate SPs. Trust value* (τ) *is represented as*  $\tau \left(℘,\rho \right)$.

(4) τ≝∀(∀℘∧∃ρ∧∃S[i])

*where (*τ*) represents the overall trust value*; ℘ *represents the self-observation on SP* (Ψ) *regarding the requested service* (S[i]); *and* ρ *represents the recommendation value regarding the quality of the same* (S[i]), *which is provided by* (Ψ). *The contribution of self-observation and recommendation is controlled by the dynamic weighting, as demonstrated in Equation (5), as follows:*

(5) $$\tau \left(\exists \rho \vee \exists ℘\right)$$

The recommendations (ρ) were selected from the list of the available recommenders by considering three factors: friendship similarity, social contact similarity, and community of interest (CoI).

**Theorem 2.**  *The trust value of the SRs for specific candidate SPs was calculated through self-observation and the recommendation of other SRs, as shown in Equation (4). Thus, we defined the theorem of the overall accurate trust assessment for the trust-based service composition, as shown in Equation (6), as follows*:

(6) $$AccurateTrustValue\left(\tau \right)\to \left(\forall CorrectRecommendation\left(\rho \right)\vee \forall AccuratSelfObservation\left(℘\right)\right)$$

**Proof of Theorem 2:**  This proved non-trivial as, although the expression in Equation (6) cannot always be evaluated as an accurate value, the received recommendation values were correct and selected based on social similarities during the recommendation calculation, and the self-observation estimation was accurate. Therefore, the theorem is only true if the recommendations are correct, and are not fabricated or maliciously modified by trust attackers, and if the self-observation is accurately estimated by the SR. □

**Theorem 3.**  *The quality of a trust-based composite service would not be very high if the SPs were selected based on inaccurate trust value estimations for each SP that provided atomic services. Inaccurate trust value estimations can result from Equation (6). Consequently, we defined the theorem using Equation (7) to represent an accurate trust value estimation, which leads to the selection of a good SP and, as a result, the best service composition*.

(7) $$⊢AccurateTrustValue\to \left(\forall CorrectRecommendation\left(\rho \right)\vee \forall AccuratSelfObservation\left(℘\right)\right)$$

**Proof of Theorem 3.**  This proved trivial as the expression defined by Equation (7) might not always be evaluated as true. This is because some recommenders might not be correct (honest) during the issuance of their recommendation, and the estimation of an SR’s self-observation may not always be accurate. Therefore, the theorem was classified as a non-universal truth as it is only evaluated as true if a recommendation is correct (honest) and an estimation of the self-observation value is accurate. □

## 4. Formal Representation of Trust System

The trust system’s goals are to estimate (computationally) the overall trust values and to monitor the behavior of SOA entities in order to drop malicious entities’ illegally obtained trustworthiness values and to reward honest entities [27]. The trust model and entity behavior model help the trust system to achieve its objectives.

### 4.1. Formal Representation of Trust Model

**Figure 1:**
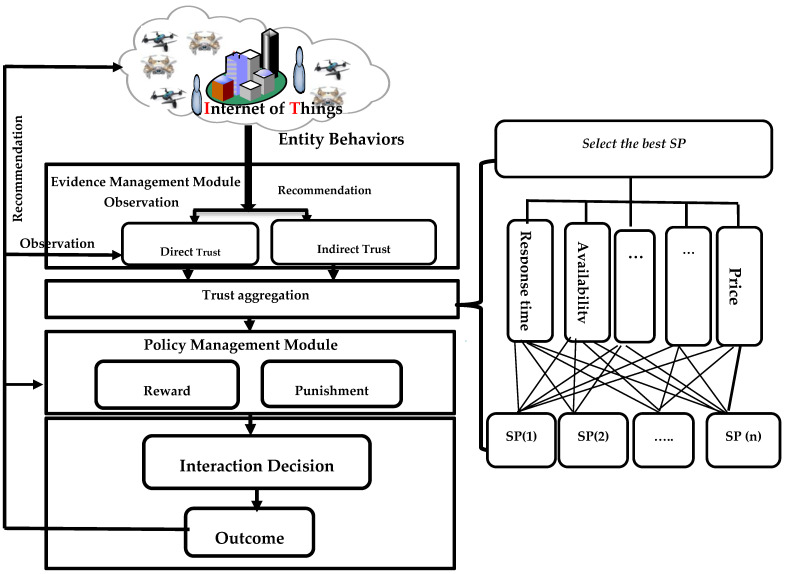
The modules of trust system for trust-based IoT.

The trust model is the brain that guides the transactions in a trust-based IoT system; therefore, the selection of SPs is performed based on the trust value. The trust and reputation subsystem helps SRs by computing the trustworthiness of the candidate SPs, as shown in Figure 1. The trust model has the following three major duties:

- Maintaining self-observation (direct trust);
- Providing recommendations (indirect trust) to other SRs;
- Computing the overall trust value.

#### 4.1.1. Recommendation (Indirect Trust)

The recommendation value, also known as the indirect trust value, is generated by a recommendation function in the trust model, whereas the recommendation function runs whenever it is called by an SR (recommender), after the completion of the requested service. The recommendation function is responsible for allowing IoT entities/SRs to exchange their experiences regarding the trustworthiness of the SPs (Guo, Chen [28]).

**Definition 4.**  *The recommender* (ρ.ip[i]) *is a SR that disseminates its experience about the SPs. For every recommendation,* ρ, *there must be at least one recommender* (ρ.ip[i]).

(8) $$\forall \rho \left(\exists_{\geq 1}rv\right)$$

**Definition 5.**  *A recommendation (*ρ*) is three-tuples—*$\rho \left(\mathrm{rv},ip[i],\Psi .ip[i],S[i]\right)$. *We can define the recommendation in HOL, as follows*:

(9) $$\forall \rho \left(\exists rv\wedge \exists recom.ip\left[i\right]\wedge \Psi .ip[i]\wedge S\left[i\right]\right)$$

*where* (recom.ip[i]) *represents the recommender;* (Ψ.ip(1)) *represents the specific SP that provides the service; and* (rv) *represents the recommendation value provided by* (recom.ip[i]) *regarding the service,* $\left(S\left[i\right]\right),$ *which is provided by the SP and is represented as* (Ψ.ip(1)).

Therefore, we used the following:

(10) $$\forall recom\;\exists \rho ↔\exists_{\geq 1}\rho .ip\left(1\right)\exists \rho .S[i]$$

where (ip) denotes the IP address of the recommender; the value (1) shows that only one IP is permitted for each recommender; (S) represents the available service; and [i] represents the service ID.

**Theorem 4.**  *For every recommendation value to be registered, there must be at least one recommender from the participating IoT entities. We defined a recommender as in Equation (11), as follows*:

(11) $$\forall \left(\rho .ip[i]\right)\to ip\left(1\right)\wedge \exists_{\geq 1}\;S\left[i\right]\wedge \exists rv$$

**Proof of Theorem 4.**  It is a simple proof because the statements in Equations (3) and (11) were always true, all recommender entities in the IoT system were associated with a single IP address, and S[i] ≥0. Therefore, the theorem is a universal truth when the recommender is active and offers a recommendation. □

#### 4.1.2. Self-Observation (Direct Trust)

Self-observation history is an important factor in trust computations; therefore, trust models must initiate and update the history of self-observation.

**Definition 6.**  *A self-observation (*℘*) is three-tuples*  $\left(ov,\Psi ,S[i]\right)$*. We can define the self-observation in HOL, as follows*:

(12) $$\forall ℘↔℘(\exists ov,\exists \Psi .ip\left(1\right)\wedge \exists S[i])$$

*where* (ip) *denotes the IP address of the SP; (1) represents that only one IP is permitted per each SP; and* S[i] *indicates that the provided service is identified as* (i).

**Theorem 5.**  *For every self-observation to be successfully registered there must be at least one transaction (experience) with the SP. Therefore, we defined a self-observation as follows*:

(13) $$\exists \Gamma \forall ℘\to \Psi .ip\left(1\right)\wedge \exists_{\geq 1}℘.S\left[i\right]\wedge \exists ℘.ov$$

**Proof of Theorem 5.**  It is a straightforward proof since the statement in Equation (13) is always evaluated as true, assuming that all the service consumer (requestor) entities in the IoT system are associated with a unique IP address, and ℘. S[i] ≥0. Therefore, the theorem is a universal truth when the service consumer is connected to the Internet, and they request and consume some services. □

### 4.2. Formal Representation of Entities’ Behavior Model

IoT entities either behave honestly or maliciously. This sub-section presents a formal representation of the behaviors of IoT entities in trust-based service composition systems.

**Definition 7.**  SPs $\left(\Psi \right)$ are a set of IoT entities, comprising honest entities and malicious/attackers. Formally, we defined SPs as a couple (𝒽,A), as shown in Equation (14), as follows:

(14)  ∀Ψ→∃𝒽∧∃A

Note that the SP can demonstrate as an honest or malicious/attacker entity.

#### 4.2.1. Formal Representation of Honest Model

The SP and SR can be modeled as honest entities based on their behavior. Honest SRs have three responsibilities: selecting the best SPs, identifying the quality of service after service completion, and sharing the experience of the service. We modeled these responsibilities as two probabilistic functions: behavioral and decision functions. These are as follows:

**Definition 8.**  *An entity behavior is four-tuple* $\left(\Gamma ,\Psi ,S[i],q_{\left(\mathbb{Q}𝕠𝕊\right)}\right)$ *and can be represented as the probability function over the quality of service*, ℚ𝕠𝕊, as follows:

(15) $$\lambda \Gamma .\lambda \Psi .\lambda S\left[i\right].q;where\left(q|q≝stisfied\right);\rho ≝\left\{\begin{array}{c} 1,\;\lambda \Gamma .\lambda \Psi .\lambda S\left[i\right].\lambda q.q):q\geq 0.5\\ 0,\;\lambda \Gamma .\lambda \Psi .\lambda S\left[i\right].\lambda q.q):q<0.5 \end{array}\right.$$

(16) $$\lambda \Gamma .\lambda \Psi .\lambda S\left[i\right].q;where\left(q|q≝ustisfied\right);\rho ≝\left\{\begin{array}{c} 0,\;\lambda \Gamma .\lambda \Psi .\lambda S\left[i\right].\lambda q.q):q\geq 0.5\\ 1,\;\lambda \Gamma .\lambda \Psi .\lambda S\left[i\right].\lambda q.q):q<0.5 \end{array}\right.$$

*where threshold is the level of untested SPs, regarding the quality of the provided service, which is defined as (satisfied, unsatisfied);* $\left(\lambda \Gamma .\lambda \Psi .\lambda S\left[i\right].q\right)$ *represents the behavior of the SPs that has been observed* (Ψ) *during the provision of the service* (S[i]) *to the entity* (Γ); *and the* q *is satisfactory if and only if the SP is trusted, otherwise, the* q *will be unsatisfactory*.

**Definition 9.**  *The decision function of a service requestor is represented as a probability function* (λΓ.λΨ.d) *over the space* (∀Ψ), *showing the chance of each entity being selected as an SP*.

#### 4.2.2. Formal Representation of Attacker Models

Trust attackers (A) are a set of entities that participate in trust-based management, but that behave maliciously, either individually or through collusion with each other to overcome the trust mechanism. Two points are associated with trusting an attacker: an attacker’s abilities and objectives. An attacker’s abilities are a series of actions that are performed during the running time of a trust-based IoT system. The objectives of the attackers were modeled as a set of punishments and rewards.

- Atomic actions

An atomic action is an action that can be carried out by an individual attacker, at a specific time during the system’s runtime. A trust attack scenario comprises a series of atomic actions. Atomic actions represent all the possible behaviors of the attackers and can be classed as passive, re-entry, and participation. These categories are defined as follows: Passivity (non-participation) is when the attacker remains passive, i.e., does not participate in the system transactions. Passive action denotes that the entity is present in the system but has not yet demonstrated itself as a requestor or service provider. Re-entry is when the attacker has behaved maliciously for some time so the trust system recognizes the attacker as an untrustworthy entity. Attackers escape from this by exiting the system to obtain a new identity and then re-entering as a newcomer. Re-entry with a new ID enables the attacker to reset its bad transactional history and, consequently, its trust records. Participation is when the attacker is willing to participate in the transactions. However, its participation involves illegal activities that redefine the behavior and decision functions to maximize their abuse of the honest SPs that are competing. These actions are illustrated as follows:

- Attacker action

The attacker action represented by (δ), involves three functions: the behavior function, the recommendation function, and the decision function. These functions are similar to honest functions. However, the attacker defines the behavior function deterministically and selects its values deliberately, instead of stochastically.

The behavior function is represented by $\left(\left(\lambda \beta .\lambda \Gamma .\lambda S\left[i\right].\lambda \delta .q\right)\right)$, and it defines the $q_{\left(\mathbb{Q}𝕠𝕊\right)}$ for the service performed, according to the malicious behavior of the attacker.

The decision function $\left((\lambda d.\lambda \Psi .\lambda S\left[i\right].{(a}_{\left(A\right)}\vee {𝒽}_{\left(H\right)}))q\right)$ is concerned with identifying the quality of the service execution if the SR(Γ) is honest, ${𝒽}_{\left(H\right)}$, and the SP is the attacker $a_{\left(A\right)}$.

The recommendation function is represented by $\left(\lambda \rho .\lambda d.\lambda \Psi .\lambda S\left[i\right].{(a}_{\left(A\right)}\vee {𝒽}_{\left(H\right)})\right)$. This function is used for modeling the recommendation values. The honest entities propagate their recommendations about the other entities based on their real experience. However, the attacker falsely propagates recommendation values about the other entities. This false recommendation function is independent of the trust model recommendation function. The attacker develops it to ruin the reputation of the honest entities. This false recommendation function of an attacker, with the action (δ), can be written as $\left(\lambda \rho .\lambda d.\lambda \Psi .\lambda S\left[i\right].\lambda \delta .{(a}_{\left(A\right)}\vee {𝒽}_{\left(H\right)})\right)$.

The actions chosen by the attackers are not consistent during the runtime. In contrast, the attacker may take different actions at different times during the runtime. To maximize their gain, the attackers carefully select the function. In some cases, a group of attackers collude with each other in coordinating their atomic actions to achieve maximum interest. For example, they may promote trust in each other, or they may abuse their competing entities for the sake of being the best in the system.

- The formal representation of rewards and punishments in the trust system

The attacker commits the atomic action to illegally obtain rewards or to incur intermediate costs (abuse). The illegal reward depends on the level of abuse aimed at the competing SPs, which provide the same service as the attacker. The objective of the attacker is to illegally achieve the maximum possible rewards. There are many types of intermediate costs incurred by attackers’ behaviors within trust-based service computing systems, such as service requests, service execution, and obtaining new IDs. The service requesting cost is the responsibility of the SR towards the SP, where

CREQ represents the cost of requesting a service; CREQ is constant.

CSRV:ℚ→𝔾, represents the cost of a service execution with quality. qℚ𝕠𝕊, which can be represented as C(q,SRV).

**Figure 2:**
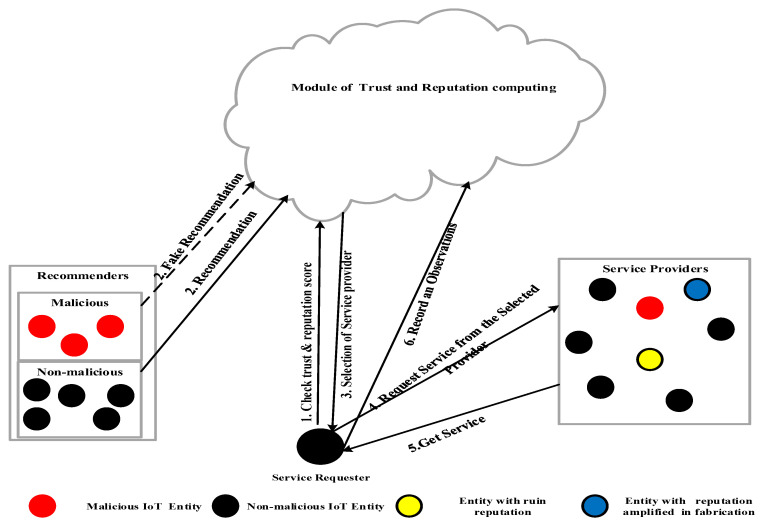
Trust-based SP selection in IoT [30].

Trust attacks either manipulate the trust value falsely or abuse the competing service provider through the use of the false trust value, as illustrated in Figure 2. The false manipulation of the trust value indicates that the attackers cheat the trust and reputation system by either falsely boosting their own trust values, known as a “self-promotion attack,” or by decreasing the trust value of the honest SPs, known as “bad-mouthing attacks” [27,29].

(17) $$RSP≝SPV\times \frac{\left(\left(\lambda d.\lambda \Psi .\lambda S\left[i\right].{(\exists a}_{\left(A\right)}\vee \exists {𝒽}_{\left(H\right)}))q\right)\right)}{\exists a_{A}\wedge \exists {𝒽}_{H}}$$

where RSP is the reward, for the self-promoting attack; SPV is the reward value of the promotion attack; and $\left((\lambda d.\lambda \Psi .\lambda S\left[i\right].{(a}_{\left(A\right)}\vee {𝒽}_{\left(H\right)}))q\right)$ is the decision regarding the chance of selecting the entity (Ψ) as an SP.

(18) $$RBM≝\left(VBM\times (1-\left((\lambda \rho .\lambda d.\lambda \Psi .\lambda S\left[i\right].\lambda \alpha .{(a}_{\left(A\right)}\vee {𝒽}_{\left(H\right)})q\right)\right)$$

$$≝VBM\times \left(1-\frac{(sum\left(a_{A}⋀{𝒽}_{H},k\right)\left(\left(\lambda d.\lambda \Psi .\lambda S\left[i\right].{(a}_{\left(A\right)}\vee {𝒽}_{\left(H\right)}))q\right)\right)}{\exists a_{A}\wedge \exists {𝒽}_{H}}\right)$$

where RBM is the reward for the slandering attack and VBM is the reward value of the slandering attack.

Abuse of the system occurs when attackers have malicious or selfish interests. In the case of malicious abuse, the attackers damage the system by executing bad-quality services.

## 5. Execution Semantics

The execution flow includes several steps, as defined in Figure 2. The home cloud of the SR aggregates the filtered recommendations and utilizes them to compute the overall trust values for each candidate in the list of SPs. Next, the decision function $\left((\lambda d.\lambda \Psi .\lambda S\left[i\right].{(a}_{\left(A\right)}\vee {𝒽}_{\left(H\right)}))q\right)$ runs to select the appropriate SP, as demonstrated in Figure 2. The SR sends a request to the SP, which responds according to its behavior function, $\left(\left(\lambda \beta .\lambda \Gamma .\lambda S\left[i\right].\lambda \delta .q\right)\right)$. After the service completion, the SR updates its self-experience (observation) and, based on this experience, sends a recommendation to the other SRs.

## 6. Performance Metrics of Trust System

This section presents the performance metrics of the trust system, namely its accuracy, resiliency, and convergence.

### 6.1. Accuracy

The difference between the predicted trust value and the most recent direct experiences of an IoT user (ground truth) is referred to as accuracy [31,32]. Trust value and accuracy of trust value were computed using Equations (19) and (20), respectively.

$$\tau_{\Gamma ,\Psi }\leftarrow \exists \omega .{\forall \tau }_{\Gamma ,\Psi }^{d}+\left(1-\exists \omega \right).{\forall \tau }_{\Gamma ,\Psi }^{r}$$

where

(19) 0≤∀ω≤1

(20) $$Accu\leftarrow MSE\left(\forall \omega \right)\leftarrow \sum_{\Psi }{\left(\exists \omega .{\forall \tau }_{\Gamma ,\Psi }^{d}+\left(1-\exists \omega \right).{\forall t}_{\Gamma ,\Psi }^{\rho }-\forall p_{\Gamma ,\Psi }^{\bar{\left(recent\right)}}\right)}^{2}$$

where less mean squared error (MSE) indicates high accuracy.

### 6.2. Resiliency

The ability of trust systems to provide accurate judgments in the presence of malicious nodes is referred to as their resilience [31,33]. The behavior of the trust model in terms of its trust accuracy against increasing malicious entities is used to calculate the system’s resiliency, as shown in Equation (21), as follows:

(21) $$Res\leftarrow \forall \left(Acc/\mu \right)$$

### 6.3. Convergence

The convergence of the trust system means the difference between the estimated trust values of an IoT node at time_1 (${ts}_{1}$) and time_2 (${ts}_{2}$) [27]. Convergence is measured by the number of times (executions) taken. Equation (22) represents how the convergence is computed, as follows:

(22) $$Conv\leftarrow {\exists ts}_{1}.\forall \tau_{\Gamma ,\Psi }-\exists {ts}_{2}.\forall \tau_{\Gamma ,\Psi }$$

## 7. Service Composition: A Case Study

The basic background of our case study was derived from the smart city system that has been used in many existing studies [27,31,34]. In our case study, when the tourist, Rania, reached city C, she was aware that city C was a smart city. She downloaded the smart traveler app on her smartphone and created an account on the social network. Moreover, she installed an augmented map—a social IoT application that is used to run near-field communication (NFC) to enable the browsing of a tag-augmented city map during sightseeing. Rania’s smartphone automatically connected with the available IoT devices, via the help of the tag-augmented map. The connection then occurred wherever the IoT devices (smart devices) encountered Rania’s smartphone in the NFC communication range. These smart devices provide information regarding food, entertainment, and transportation services, and they enable ticket purchasing. The system allowed for Rania to instruct her smartphone to dynamically make decisions about the service selection. The selection process depends on direct observation (new information) and recommendations from nearby IoT devices. Responding to Rania’s request, her smartphone performed the following three tasks: (i) it collected sensing information, which was gathered from the physical environment through self-observation and recommendation; (ii) it used the collected information in the formulation of a service composition plan; and (iii) it invoked suitable services to fit Rania’s service request. The aforementioned processes were then composed into a workflow plan by the augmented-map tourist service composition app, which ran on Rania’s smartphone.

**Figure 3:**
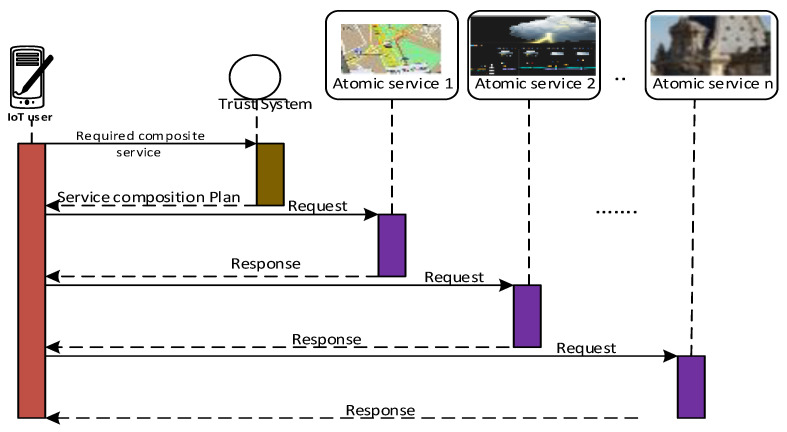
Trust-based service composition.

**Figure 4:**
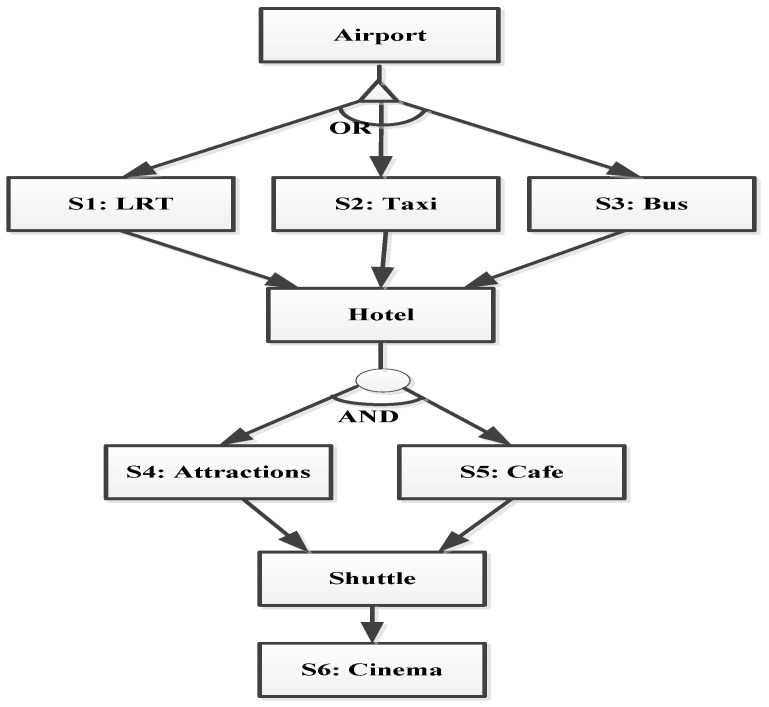
Trust-based service composition (tourist plan).

Figure 3 demonstrates Rania’s travel planning, in which there were different activities (atomic services) included in her request. The trust-based service composition application, which ran on Rania’s smartphone, selected the best and most trustworthy SPs to provide the required atomic service, as specified in the workflows. Figure 4 presents six sub-services (atomic), which were organized and executed based on three types of workflow structures: selection, parallel, and sequential. Each atomic service had multiple SP candidates. The overall trustworthiness value of this service composition application was computed recursively. In particular, the trustworthiness of a composite service depends on the structure that connects its two atomic services. In the workflow structure shown in Figure 4, the sequential structure forms the overall service composition plan by connecting the three groups of atomic services, in which a selection structure connects one atomic service inside the group and a parallel structure connects the other atomic service inside the group.

## 8. Conclusions

In this article, we represented trust systems in the context of trust-based service composition by using the HOL formal method. The aim of this study was to provide a better understanding of trust-based service composition. We presented the crucial component of the trust system formally by concentrating on the whole trust system’s responsibility, the trust computation model, the attackers’ model, and the honest entities’ model. The formal methods assist the designer of the service composition applications in understanding the trust system and in avoiding the selection of bad SPs, which may not be considered during the design and development phases. Thus, we utilized the HOL formal method to represent the trust-based service composition, with a focus on crucial issues such as SP selection. Furthermore, we highlighted the performance metrics of the trust system in trust-based service composition. In future work, we plan to consider further details, such as centralized and decentralized trust systems, and smart devices’ mobility and their impact on trust-based service composition in IoT environments.

## Figures and Tables

**Table 1:** Related studies.

Study	Trust-Based	Model/Logic	Application Domain
This study	Yes	Higher-order, logic-based	IoT
A hybrid formal verification approach for QoS-aware multi-cloud service composition [21]	No	Multi-labeled transition systems-based model, checking, and Pi-calculus-based process.	Cloud computing
Formal verification for web service composition: a model-checking approach [22]	No	Temporal logic and model-checking approach for verifying service composition.	General
Semantic web service composition Using Formal Verification Techniques [23]	No	Semantic matchmaking and formal verification techniques: Boolean satisfiability solving and symbolic-model checking.	General
Formal verification of Service composition in pervasive computing environments [24]	No	Labeled transition system, by transforming concurrent regular expressions into Finite State Process notation.	General

**Table 2:** Some logical symbols of HOL.

Symbols	Meaning	Explanations
∀	For all	$\forall_{x}\;means\;for\;all\;probable\;values\;of\;x$
∃	Exist	$\exists_{x}\;means\;existance\;of\;a\;value\;for\;x$
⋀	Conjunction	(X∧Y) is true if and only if X is true and Y is true
∨	Disjunction	(X∨Y) is true if either one of X is true or Y is true.
→	Implication	$X\to \left(Y\wedge Z\right)$ states the truth of X if and only if Y and Z are true. Similarly, the statement X→(Y∨Z) states the truth of X if either Y or Z are true.
↔	Bi-conditional	(X↔Y) asserts X if and only if Y
⊣	Negation	The statement (⊣X) states that X does not yield X
≝	Equality	$\left(X≝Y\wedge Z\right)\;states\;that\;the\;truth\;of\;Y$ and the truth of Z equals X, by definition.
$x_{\left(Z\right)}$	Variable x	x belongs to set Z
λx.f(x)	Lambda function	Nameless function of x with function definition given by the expression f(x)
$\left(\lambda x.f\left(x\right)\right)c$	Replacement statement	Replacement of x in expression f(x) , resulting in f[x≔c]
sum(0,k)(λx.f(x))	Summation function in the range of 0 to k	$\sum_{x=0}^{k}f(x)$

**Table 3:** The variables of trust and reputation system.

Category	Symbol	Description
Trust model	∑	Trust score (history)
	⊕	Update function
	Ρ	Recommendation function
Honest model	Τ	Trust function
	𝒽	Honest entity
	Β	Behavioral function
	D	Decision function
Attackers/malicious model	A	Attacker
	$𝔸\mathbb{C}=\left\{\left(\beta ,d,\rho \right),Passive,NewID\right\}$	Atomic action
	$\left\{C_{REQ},C_{SRV}^{q},C_{newID},G_{SRV}^{q}\right\}$	Intermediate cost
	$\left\{G_{PR},G_{SL},G_{DM}^{q}\right\}$	Intermediate gain

## Data Availability

Not applicable.
